# Pathogenesis of Musculoskeletal Deficits in Children and Adults with Inflammatory Bowel Disease

**DOI:** 10.3390/nu13082899

**Published:** 2021-08-23

**Authors:** Lewis Steell, Stuart R. Gray, Richard K. Russell, Jonathan MacDonald, John Paul Seenan, Sze Choong Wong, Daniel R. Gaya

**Affiliations:** 1 Institute of Cardiovascular and Medical Sciences, University of Glasgow, Glasgow G12 8QQ, UK; lewis.steell@glasgow.ac.uk (L.S.); Stuart.Gray@glasgow.ac.uk (S.R.G.); 2 Department of Paediatric Gastroenterology, Royal Hospital for Sick Children, Edinburgh EH16 4TJ, UK; Richard.russell@nhslothian.scot.nhs.uk; 3 Department of Gastroenterology, Queen Elizabeth University Hospital, Glasgow G51 4TF, UK; Jonathan.macdonald@ggc.scot.nhs.uk (J.M.); johnpaul.Seenan@ggc.scot.nhs.uk (J.P.S.); 4 Department of Paediatric Endocrinology, Royal Hospital for Children, Glasgow G51 4TF, UK; jarod.wong@glasgow.ac.uk; 5 Department of Gastroenterology, Glasgow Royal Infirmary, Glasgow G4 0SF, UK

**Keywords:** inflammatory bowel disease, Crohn’s disease, bone, muscle, osteoporosis, inflammation

## Abstract

Musculoskeletal deficits are among the most commonly reported extra-intestinal manifestations and complications of inflammatory bowel disease (IBD), especially in those with Crohn’s disease. The adverse effects of IBD on bone and muscle are multifactorial, including the direct effects of underlying inflammatory disease processes, nutritional deficits, and therapeutic effects. These factors also indirectly impact bone and muscle by interfering with regulatory pathways. Resultantly, individuals with IBD are at increased risk of osteoporosis and sarcopenia and associated musculoskeletal morbidity. In paediatric IBD, these factors may contribute to suboptimal bone and muscle accrual. This review evaluates the main pathogenic factors associated with musculoskeletal deficits in children and adults with IBD and summarises the current literature and understanding of the musculoskeletal phenotype in these patients.

## 1. Introduction

Musculoskeletal deficits are among the most common extra-intestinal manifestations and complications of inflammatory bowel disease (IBD), especially in those with Crohn’s disease (CD). Specifically, patients with IBD are at an increased risk of osteoporosis, a systemic disorder characterised by low bone mass and deterioration of bone microarchitecture, with consequent increased risk of fractures throughout their lifetime [1]. In adults without underlying chronic disease, osteoporosis is primarily an age-related disease of abnormal bone metabolism, whereby excessive bone resorption results in net bone loss. This is especially common in postmenopausal women [2]. In contrast, secondary osteoporosis relates to bone loss that occurs because of other factors, such as chronic disease, exposure to medications that alter bone metabolism, or impaired mobility [3]. In growing children with chronic disease, the underlying pathophysiology of secondary osteoporosis is mainly due to inadequate skeletal development, rather than bone loss per se [4]. Poor bone accrual during childhood may lead to the attainment of lower peak bone mass as adults. Specifically in paediatric inflammatory diseases such as IBD, multiple factors including underlying disease activity, undernutrition and exposure to systemic glucocorticoids may contribute to poor bone mass accrual. As peak bone mass is a critical determinant of future skeletal morbidity risk, these individuals may incur an increased risk of osteoporosis later in life.

**Figure 1:**
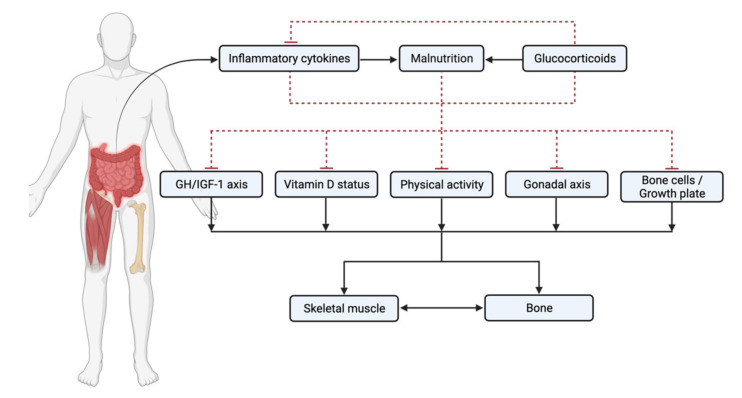
Factors associated with musculoskeletal deficits in children and adults with inflammatory bowel disease (IBD). Solid line = promoter; dashed line = inhibitor.

On top of the well-established bone defects, emerging studies in the paediatric and adult IBD show that muscle mass and function are low compared to healthy peers [5,6]. These muscle deficits may contribute to overall disease activity and are adversely associated with responsiveness to IBD therapies [7,8] and postoperative morbidity [9,10]. Throughout the lifespan, it is known that there is close bi-directional crosstalk between muscle and bone [11]. In IBD, factors including chronic inflammation, nutritional deficits, and use of long-term glucocorticoids have direct and indirect adverse effects on both muscle and bone (Figure 1). This review aims to summarise the latest understanding of these factors and their associations with musculoskeletal deficits in children and adults with IBD, primarily focusing on CD.

## 2. Pathogenesis of Musculoskeletal Deficits in IBD

### 2.1. Inflammation/Cytokines

Pro-inflammatory cytokines perpetuate gastrointestinal inflammation and tissue destruction in IBD while regulating extra-intestinal manifestations of the disease, including bone and muscle deficits. The role of inflammation in the pathogenesis of skeletal deficits in IBD has been highlighted by reduced bone mass in newly diagnosed cohorts of treatment naïve paediatric IBD patients [12,13,14] and the report of osteoporotic vertebral fracture as the presenting feature of CD in an adolescent [15]. 

Several cytokines are known to be chronically elevated in the serum of individuals with IBD, such as TNF-α, IL-1β, and IL-6 inhibit osteoblast function (responsible for bone formation) and promote osteoclastogenesis (responsible for bone resorption) [16,17,18]. TNF-α appears to be the master regulator of bone loss in IBD as it promotes expression of receptor activator of nuclear factor κB ligand (RANKL) from osteogenic cells and independently interacts with RANKL as a potent stimulator of osteoclastogenesis, via the nuclear factor kappa B (NF-κB) signaling pathway [19,20]. In what appears to be an attempt to maintain bone homeostasis, the decoy RANK receptor, osteoprotegerin (OPG), which inhibits osteoclastogenesis, has been found to be elevated in the plasma of IBD patients [21]. However, this mechanism appears insufficient to reverse RANKL and TNF-α mediated bone resorption, as OPG levels were inversely associated with bone density [21]. During a disease flare, elevated serum RANKL was observed in CD compared to healthy controls, highlighting the influence of inflammation on the RANKL/OPG pathway in perpetuating bone resorption [17]. Inflammatory cytokines also adversely affect bone formation in IBD. Serum from treatment naïve CD patients inhibited osteoblast function in vitro, indicating a biochemical component of CD that negatively regulates bone formation [22,23], with other studies demonstrating the pivotal role of IL-6 [24]. In a murine model of colitis (Piroxicam treated IL-10 knock out mice), the elevation of gut expressed inflammatory cytokines, including TNF-α, IL-1, IL-6 and IL-17A were associated with a reduction in circulating levels of bone formation markers, increased bone resorption, and reduction in trabecular thickness at the tibia [25]. The interaction between activated immune cells and cytokines with bone cells and metabolism is a continually evolving area of research. Several cytokines upregulated in IBD have been found to influence bone turnover in vitro, in animal studies, and in other inflammatory pathologies (reviewed elsewhere [26]).

TNF-α and IL-6 are also key regulators of muscle loss in CD, a pathway shared with aging. Circulating TNF-α and IL-6 are increased in healthy elderly adults and are negatively associated with muscle mass and function [27]. These and other pro-inflammatory cytokines are elevated in the serum of quiescent IBD compared with controls [5,23,28]. While cytokines play a key role in normal skeletal muscle homeostasis and regeneration, in the context of dysregulated immune function as that in CD, they moderate and contribute to skeletal muscle loss. Increased levels of TNF-α and IL-1β result in the activation of NF-κB pathways to accelerate protein degradation [29] and decrease expression of pro-myogenic factor, MyoD [30]. Excessive TNF-α may also reduce the regenerative capacity of skeletal muscle satellite cells via inflammation-induced mitogen-activated protein kinase signalling that leads to epigenetic alterations and inhibition of myogenesis [31,32]. Ultimately, these pathways will promote inflammatory mediated muscle loss in IBD with reduced muscle mass and function. 

### 2.2. Nutrition

Individuals with IBD are at increased risk of sub-optimal nutritional status which can itself impact bone and muscle. The aetiology of poor nutritional status in IBD is multifactorial and includes disease activity associated with anorexia, exclusion diets, medication-induced nausea, pain from strictures, and malabsorption from both active disease and small bowel resections. However, both undernutrition and overnutrition are known to be associated with adverse effects on the musculoskeletal system. Historically, rapid and unsolicited weight loss was a presenting symptom of disease in both CD and ulcerative colitis (UC), indicative of nutritional deficiency. For example, malnutrition as defined by a body mass index (BMI) Z-score < −2 SDS was observed in approximately 30% of paediatric CD patients at diagnosis in one prospective study, reducing to 15% at the long-term follow-up [33]. Some historical estimates suggest 60–80% of CD and 30–60% of UC patients present with undernutrition at disease diagnosis [34,35,36]. In paediatric CD, low weight Z-score was associated with low height Z-score, and the amelioration of nutritional status after anti-TNF-α therapy was associated with improved linear growth irrespective of pubertal progression [37]. 

Dietary intake and composition are intrinsically linked to disease management in IBD, as exemplified by the efficacy of exclusive enteral nutrition (EEN) in inducing remission and mucosal healing in CD [38,39] and other potential therapeutic dietary interventions [40]. A proportion of patients with IBD may develop food aversions or follow self-imposed exclusionary diets to prevent perceived exacerbation of disease symptoms [41,42,43]. This could lead to inadequate dietary intake and subsequent micro and macronutrient deficiencies, which have been observed in adults with quiescent disease [44]. Dietary calcium intake was found to be lower in patients with IBD from a dietary questionnaire study, due to perceived lactose intolerance [45]. In another study, low calcium intake was associated with reduced femoral neck bone density in a cohort of young adults with CD [46]. Furthermore, pro-inflammatory cytokines have been shown to directly interfere with appetite hormones which may reduce hunger and subsequently dietary intake in IBD [47]. 

Inadequate dietary intake has been reported in patients with both active and inactive IBD [42,48,49,50]. During active phases of the disease, elevated metabolic expenditure due to perpetual immune activation may also contribute to malnutrition, further compounding nutritional deficits and contributing to musculoskeletal loss. Separate from inadequate dietary intake, intestinal malabsorption can also be a particular concern to some patients with CD. Patients with significant small bowel CD can present a particular challenge to clinicians; active stricturing small bowel disease will more often present with nutritional issues as malabsorption and post-prandial pain (leading to food avoidance) are additive in their detrimental effects. Moreover, after repeated resective surgeries (more of a historical issue that now rarely occurs) such patients can become dependent on parental nutrition and require lifelong close follow-up and monitoring to prevent the many complications of undernutrition in this setting.

Contrary to the typical undernutrition observed in IBD, clinicians managing these patients are encountering an increasing frequency of patients who are overweight and obese, in line with the obesity epidemic present in the general population. Prolonged systemic glucocorticoid therapy by itself could of course contribute to weight gain. This creates a separate issue of overnutrition that may contribute to bone and muscle defects. Recent studies show that the prevalence of obesity among adults with IBD is estimated at 10–50% [51,52,53], whereas 10–30% of paediatric IBD patients are obese at diagnosis [54,55]. Obesity is associated with poor bone microarchitecture relative to body weight [56] thought in part to be driven by increased levels of inflammatory adipocytokines. While obesity is generally associated with slightly increased muscle mass, muscle quality is adversely affected by the associated accumulation of intramuscular fat, which leads to poor muscle function and is a risk factor for musculoskeletal morbidity [57]. 

BMI is generally used as a clinical marker of nutritional status. However, it is an insensitive measure of adiposity as abnormal body composition, characterised by low lean mass and maintained or increased fat mass, is common in paediatric and adult IBD [58]. Resultantly, ‘normal’ BMI can also be falsely reassuring as increased adiposity, especially visceral adiposity, despite BMI in the normal ranges have been described in IBD [59,60]. Visceral adipose tissue expresses pro-inflammatory adipocytokines such as TNF-α, IL-1β, and IL-6 that may further perpetuate systemic inflammation and contribute to bone and muscle loss in CD [28]. The potential role of increased visceral adiposity on disease activity itself requires further research but may highlight the necessity of avoiding overnutrition in IBD patients. In the clinic, estimates of fat mass and lean mass using skin calipers, mid-arm circumference and, if available, bioimpedance may help identify specific areas requiring action. Use of these and the development of novel outcome measures that accurately reflect nutritional status in IBD require further research as the nutritional status may be an important confounder of musculoskeletal health.

### 2.3. Glucocorticoids

Systemic glucocorticoids are commonly administered as first-line induction therapy for moderate to severe IBD in adult and paediatric patients. Glucocorticoids are effective in achieving clinical remission in IBD; however, they are associated with systemic toxicity, including profound and devastating effects on bone and muscle. Chronic glucocorticoid exposure can lead to muscle atrophy and glucocorticoid-induced osteoporosis with an increased risk of fragility fractures [61,62,63]. In children, glucocorticoid exposure can also lead to delayed pubertal development and inhibition of linear growth [64,65]. Given that linear growth and pubertal development are also major drivers of bone development during childhood, it is not surprising that factors that inhibit growth and puberty lead to inadequate bone accrual. 

In adults, glucocorticoid-induced bone and muscle loss occur in a rapid, dose-dependent manner. Glucocorticoids induce preferential loss of metabolically active trabecular bone [66], highlighted by the increased risk of vertebral fractures in glucocorticoid treated populations even with short-term exposure. Primarily, glucocorticoids-induced bone loss occurs via protracted reduction of bone formation, via inhibition of osteoblast function [61]. They have also been observed to induce apoptosis in osteoblasts and osteocytes [67]. An initial increase in bone resorption is also observed as glucocorticoids stimulate increased expression of RANKL from osteoblastic cells while downregulating OPG expression [68]. Additionally, glucocorticoids reduce the capacity of mature osteoblasts to synthesise type I collagen, leading to a reduced volume of the bone matrix for mineralisation [69]. Extra-skeletal effects of glucocorticoids with an impact on the skeleton include impaired intestinal absorption of calcium and phosphate, impaired vitamin D metabolism, and increased renal calcium excretion, which may lead to secondary hyperparathyroidism [61].

In children, glucocorticoids directly inhibit linear growth via peripheral and central mechanisms which in turn can impair bone development. Glucocorticoids cause local insensitivity to growth hormone (GH) and insulin-like growth factor 1 (IGF-1) at the growth plate by disrupting GH/IGF-1 receptors in chondrocytes [70]. Glucocorticoids also inhibit IGF-1 signalling which impedes proliferation and upregulates apoptosis locally in chondrocytes at the growth plate [71]. Chronic exposure to glucocorticoids in children can also contribute to delayed puberty via the development of hypogonadotropic hypogonadism due to gonadotrophic releasing hormone (GnRH) suppression [72]. The use of glucocorticoids in paediatric IBD patients is therefore avoided where possible, or very closely monitored and tapered as quickly as is clinically viable to minimise the potential for growth or pubertal disturbance.

The catabolic effects of glucocorticoids within the muscle may also negatively influence bone. The muscle wasting properties of glucocorticoids can be observed as little as seven days after initiation of therapy [73]. Glucocorticoids regulate muscle protein balance by inhibiting muscle protein synthesis and accelerating proteolysis, resulting in net catabolism. Exogenous glucocorticoids inhibit muscle protein synthesis through reduced expression of hepatic and muscle IGF-1 and upregulate skeletal muscle atrophy-related genes including muscle RING finger 1 (MuRF1) and muscle atrophy F-box (MAFbx) [74,75]. 

All the above has led to cautious and limited use of glucocorticoids in IBD and early institution of steroid-sparing measures. Indeed, avoidance of repeated courses of glucocorticoids is a key performance indicator in the audit of UK IBD units [76]. While there is no doubt that even a short course of glucocorticoids has a negative impact on bone, it must be emphasised that inflammation itself is also a driver of bone loss or attenuation of bone accrual. Therefore, if glucocorticoid remains the only therapeutic strategy that may lead to induction of remission in patients with moderate or severe disease, this option should be used for as short a duration as possible. 

### 2.4. Vitamin D

Vitamin D has pleiotropic physiological functions but is primarily recognised for its role in the regulation of calcium homeostasis and bone metabolism. Low levels of vitamin D can lead to secondary hyperparathyroidism, whereby excessive parathyroid hormone expression accelerates bone turnover with subsequent reductions in bone strength. Additionally, vitamin D plays an integral role in both innate and adaptive immunity (extensively reviewed elsewhere [77]), including in the maintenance of intestinal barrier function and inhibition of pro-inflammatory cytokine expression through suppression of T-cell activation. Resultantly, low circulating vitamin D levels may indirectly affect skeletal outcomes via the loss of these immunomodulating functions. In IBD, several factors can contribute to vitamin D deficiency, including intestinal malabsorption, poor dietary intake, and lack of sunlight exposure (due to both inactivity during disease flares and following medical advice on avoidance while taking immunosuppressive medications). Low levels of vitamin D are more common in IBD compared to the general population and more common in CD compared to UC [78]. Prior studies have observed vitamin D insufficiency (serum 25-hydroxyvitamin D [25-OHD] < 50 nmol/L) or deficiency (<25 nmol/L) in 30–70% of adult IBD patients [5,44,78,79,80].

While there is growing interest in the link between vitamin D and skeletal muscle mass and function, a direct causal role for low vitamin D in IBD-associated musculoskeletal pathology has not been clearly established. To date, no studies have investigated the direct role of vitamin D status on skeletal muscle of patients with IBD. However, low serum vitamin D3 was associated with impaired activation of muscle protein synthesis pathways in the skeletal muscle of young adults with CD [81]. Additionally, muscle function improved in children with IBD after long-term vitamin D supplementation, suggesting a potential role that warrants future investigation [82]. One study found levels of the active form of vitamin D (1,25-dihydroxy vitamin D) to be higher in the serum of CD patients compared to UC, and to be inversely associated with bone density in CD [83]. The authors postulated that conversion of 25-OHD to 1,25-dihydroxy vitamin D may be greater in CD to support immune function and the observed association with skeletal outcomes possibly reflects the higher inflammatory burden of CD rather than a specific skeletal effect. Nevertheless, low serum 25-OHD was previously associated with accelerated bone turnover in a cross-sectional study of adults with CD [84] and was reported as a risk factor for low bone density in another cohort [85]. Other studies in IBD, however, have failed to report an association between vitamin D status and bone density [86]. Still, improvements in bone density have been observed after long-term vitamin D and calcium supplementation in children [82] and adults [87] with IBD. A treatment effect of vitamin D is unlikely to be observed if the population is already vitamin D replete. In clinical practice, optimization of vitamin D levels is paramount in those with severe disease and/or prolonged therapy with glucocorticoids.

### 2.5. GH/IGF-1

Chronic inflammatory diseases such as IBD can interfere with the systemic GH/IGF-1 axis. Inflammatory cytokines, glucocorticoid exposure, and poor nutrition all adversely affect GH/IGF-1 with subsequent impacts on health outcomes. The GH/IGF-1 axis is a primary regulator of linear skeletal growth and muscle accrual during childhood and puberty [88]. The GH/IGF-1 axis also plays a critical role in the maintenance of muscle-bone outcomes across the adult lifespan. GH stimulates the proliferation of osteoblasts and chondrocytes [89,90], promotes bone formation in mature osteoblasts [90] and induces local IGF-1 expression at the growth plate [88,91], all of which stimulate linear growth. In skeletal muscle, the anabolic actions of GH are mediated by systemic and local IGF-1, which stimulate muscle protein synthesis and reduce protein degradation [92]. 

In paediatric CD, faltering linear growth is a concern that often precedes gastrointestinal symptoms and may be linked to a range of systemic abnormalities in the GH/IGF-1 axis, including a relative state of hormone insensitivity or hormone insufficiency [93]. Systemic inflammation, use of glucocorticoids, and poor nutrition inhibit multiple aspects of the endocrine GH/IGF-1 axis [94]. Pro-inflammatory cytokines such as IL-6 and TNF-α reduce IGF-1 through inhibition of GH signal transduction [95] and downregulation of GH receptor expression [96], respectively, in hepatocytes. IL-6 also increases proteolysis of insulin-like growth factor binding protein3 (IGFBP3), which inhibits IGF-1/IGFBP3 complex formation and leads to increased IGF-1 clearance [97]. While inflammation, poor nutrition, and the use of glucocorticoids can have a negative impact on the systemic GH/IGF-1 axis, it is also now well known that such factors can also play a role in growth failure by a direct impact on the growth plate. 

Generally, normal levels of GH secretion with reduced IGF-1 and IGF binding proteins (IGFBPs) in paediatric CD have been reported in published studies, which reflects a state of systemic GH insensitivity [98]. However, as mentioned, a range of different abnormalities in GH/IGF-1 have been observed in these growing children [93]. Circulating IGF-1 and IGFBP3 levels are also lower, especially during active disease, in adults with CD, although partially normalised after treatment with glucocorticoids in multiple reports [99,100]. To the best of our knowledge, there are no published studies evaluating the impact of the abnormal GH/IGF-1 axis on musculoskeletal health outcomes in adults with IBD. In a preliminary study, recombinant human growth hormone injections for moderate to severe CD in adults were associated with lower disease activity index at four months [101]. 

### 2.6. Delayed Puberty and Sex Steroid Deficiency

Sex steroids are essential for bone and muscle accrual in adolescents and maintenance of these outcomes in adults. Puberty is a critical period of bone and muscle growth that is moderated by the associated surge in sex steroid production, accompanied by an increase in the amount and amplitude of GH. Bone mass approximately doubles between the pre-pubertal and young adult age, and 90% of peak bone mass will be attained by late adolescence. Oestrogen is essential for the maturation and mineralisation of bone and the closure of epiphyseal growth plates in both sexes [102], and oestrogen deficiency in adults is associated with rapid bone loss, as observed in postmenopausal women [103]. The actions of oestrogen in bone are manifold, including the proliferation of osteoblast precursors, stimulating bone formation in mature osteoblasts, and inhibiting osteoclastogenesis [102,104,105]. Androgens also have important roles in skeletal development and maintenance and are responsible for the sexual dimorphism of bone. Androgens promote osteoblast proliferation and upregulate androgen receptors in growth plate osteoblasts, stimulate longitudinal and radial bone growth, and preserve adult bone directly via interaction with androgen receptors and indirectly via aromatization to oestrogens [106,107]. Deficiency of sex steroids is associated with reductions in bone strength and increased risk of fracture in both sexes [108,109].

The insidious onset of paediatric IBD typically manifests in the pre or early pubertal age and can delay the timing of puberty. Delayed puberty is more common among children with CD and less so in those with UC. Adolescents with CD who fail to achieve disease remission or who experience frequent disease relapses are at the greatest risk of pubertal delay. Studies have used various maturational markers to assess pubertal delay in paediatric CD, including bone age [98], age at menarche [110], and age at pubertal growth spurt [111]; all of whom showed some delay compared to healthy controls or published normative data.

Similarly, nutritional deficits, inflammatory cytokines, and prolonged use of glucocorticoids are implicated in the pathogenesis of pubertal delay in adolescents with CD [112]. Chronic undernutrition is associated with reduced fat mass, with subsequently reduced levels of leptin [113], which is an essential hormone for the advent of puberty. In studies using animal models of CD, however, reduced leptin was not the sole regulator of delayed puberty in CD and pro-inflammatory cytokines were found to have a direct role, likely through their inhibitory actions on gonadotropin secretion [114]. This hypothesis was supported by a subsequent study in children with active CD who were treated with anti-TNF-α induction therapy. Anti-TNF-α therapy led to a reduction of pro-inflammatory cytokines and increasing levels of circulating sex hormones and gonadotropins, independent of changes in body composition [115].

Few studies have been conducted to assess sex steroid deficiency in adults with IBD. Serum testosterone has been reported lower in men with CD compared to healthy controls, but clinically relevant testosterone deficiency was uncommon [116,117]. Low levels of oestradiol compared to controls have also been reported, although no association between oestradiol and bone density or turnover markers was observed [117]. In clinical practice, assessment of gonadal function (i.e., sex steroids and gonadotrophins) is important in adults with IBD who present with fragility fractures and should be part of the routine assessment in the metabolic bone clinic, as recommended by the International Osteoporosis Foundation [118]. Sex steroid replacement therapy (with oestrogen or testosterone) may be indicated if evidence of hypogonadism is identified.

### 2.7. Low Muscle Mass

Muscle and bone health are intricately related across the lifespan and their inherently linked physiology means one should not be considered independently of the other. The appropriate muscle outcome measure that can be incorporated into routine clinical care is still unknown. Low muscle mass, defined as a low skeletal muscle index on lumbar CT scan, has been reported in >50% of adult CD patients at diagnosis [119] and during a disease flare [10]. Similarly, using DXA, 60% of CD patients in remission had low muscle mass and 91% of these had concurrent osteopenic bone density T-scores [120]. We recently reported approximately 20% lower muscle cross-sectional area of the mid-thigh in young adults with childhood-onset CD, despite mild disease activity, compared to healthy controls [5]. Despite observing no deficits in trabecular microarchitecture between CD and controls in our study, muscle area was positively associated with trabecular bone volume in the CD group. Impaired activation of muscle protein synthesis pathways in young adults with CD was observed in one mechanistic study that may partly explain the observed muscle deficits [81]. Despite this, however, it remains unclear whether muscle deficit is inherent to CD or sequelae of inadequate disease control and associated lifestyle factors.

In paediatric IBD, deficits in muscle mass Z-scores are common among newly diagnosed patients [6,12,14,121,122] and those with established disease [12,123,124]. Effective control of underlying disease, however, leads to only partial improvement in muscle mass Z-scores and persistent deficits have been observed after EEN [121] and anti-TNF-α therapy [123,124]. While there are no longitudinal studies of muscle function in paediatric IBD with follow-up from adolescence to adulthood, our recent study of low muscle mass in well-controlled young adults with childhood-onset CD [5], together with the published data in paediatric CD, leads us to believe that muscle deficits may persist long-term. 

### 2.8. Physical Inactivity 

Physical activity and exercise regulate bone largely via the influence of mechanical loading. Bone tissue is sensitive to its biomechanical environment and adapts accordingly to mechanical loading or disuse, as described by Frost’s Mechanostat theory [125]. Bone adaptation occurs secondary to mechanical loading to optimise bone mass and geometry in relation to functional demands. In the context of reduced or absent mechanical loading, rapid bone loss occurs in adults and bones fail to adequately develop in children [126,127]. 

Low levels of habitual physical activity and exercise are highlighted in observational data from self-reported [128,129,130] and accelerometry [131,132] studies in children and adults with IBD. In one accelerometry study, however, similar adherence to the recommended guidelines of 60 min per day of moderate to vigorous physical activity (MVPA) between adolescents with IBD and healthy age- and sex-matched controls was reported (31% vs 38%, respectively) [133]. However, due to underlying disease and other contributing risk factors, low-level adherence to MVPA is likely to disproportionately affect the musculoskeletal health of those with IBD. Individuals with IBD experience several barriers that limit their participation in physical activity and exercise, including chronic fatigue, abdominal and joint pain, and increased urgency [128,129,134]. Negative associations between disease activity and inflammatory markers with physical activity have also been reported in CD [131,134]. Few studies have assessed the associations between physical activity and musculoskeletal outcomes in IBD, although one observed a positive association between accelerometry measured MVPA and bone density in adolescents [135]. 

The potential role of exercise in alleviating musculoskeletal burden in IBD has been little studied to date. In two exercise intervention studies, twelve months of low-impact core strengthening exercise [136] and six months of combined resistance and impact training [137] elicited mild improvements in bone density outcomes in adults with quiescent or mild CD. The latter resistance-based exercise intervention also successfully increased muscle function parameters by up to 50% in CD [137]. No studies have yet evaluated the effects of an exercise intervention on musculoskeletal outcomes in paediatric IBD, despite its potential to augment bone and muscle development. Research into incorporating physical activity as part of the care package of patients with IBD, especially early on following the diagnosis, is needed. 

## 3. Clinical Studies of Bone Health in IBD

Several imaging studies have been employed attempting to elucidate the musculoskeletal phenotype in different IBD populations. The majority of published studies in IBD have employed dual-energy absorptiometry (DXA) for the assessment of bone density. Given that short stature and delayed growth and puberty are more common in paediatric chronic diseases like IBD, areal bone density will under-estimate bone mass and therefore the need for appropriate size adjustment is now recognised. As mentioned, this is performed by volumetric adjustment of spine bone density data or adjusting for height, lean mass, or bone age for bone density data at other sites [138,139,140]. Peripheral quantitative computed tomography (pQCT) gives insight into the volumetric bone density of the trabecular and cortical bone and is not size-dependent. In addition, information on bone geometry is provided. High-resolution imaging modalities like high-resolution peripheral quantitative computed tomography (HR-pQCT) and MRI provide more detailed information on bone microarchitecture, which previously could only be attained from bone biopsies.

The greater risk of adverse musculoskeletal health in CD compared to UC is reflected in the preponderance of CD patients included in published bone imaging studies. The complexity of contributing factors to bone health is highlighted by some conflicting data in adult studies, whereas the skeletal phenotype appears more consistent in studies of paediatric IBD. Table 1 and Table 2 summarise published studies of bone phenotype in paediatric and adult IBD, respectively. 

The currently understood skeletal phenotype in paediatric IBD, predominantly in paediatric CD, is characterised by deficits in cortical bone geometry with low volumetric bone density (meaning adjusted for size) but maintained or slightly elevated cortical volumetric bone density. Abnormalities are evident at diagnosis in paediatric IBD, with deficits in total areal bone density [6,13,14,143], trabecular volumetric bone density [6,12,122], and cortical thickness [6,12,14,122] observed in DXA and pQCT studies. Adverse skeletal outcomes have been demonstrated independently of linear growth or pubertal delay in studies that reported low DXA and pQCT Z-scores in IBD when adjusted for height [6,86], bone size [58,122], or bone age delay [142]. Prospective studies have failed to report normalization of bone deficits after long-term follow-up in paediatric CD patients [6,122] or in those treated with EEN [121] or anti-TNF-α [123,124,141], suggesting that even with modern treatment strategies low-grade chronic inflammation may still be present. Currently, little is known regarding microarchitectural parameters in paediatric IBD, as no in vivo high-resolution imaging studies have been performed exclusively in this population. However, one study analysed trans-iliac bone biopsies in a small cohort of newly diagnosed IBD patients (n = 20) and found normal trabecular bone volume despite cortical deficits, inhibited bone turnover activity, and low volumetric bone density [14].

In adults with IBD, imaging studies of bone health have been almost exclusively conducted using DXA, excluding two high-resolution pQCT (HR-pQCT) [144,145] and one high-resolution MRI study from our own group [5]. The skeletal phenotype in adults with IBD is less clear, as some conflicting data exists between studies which may relate to the heterogeneity of the patient population studied, including age at assessment, duration of disease, treatment strategies (particularly the frequency of use of glucocorticoids therapy), and skeletal sites studied. Nonetheless, most study data indicate low areal bone density, particularly at the lumbar spine and hip regions [146,147,149,151]. This was further highlighted in a recent meta-analysis that reported mean difference in areal bone density Z-scores of -0.52 (95%CI: −0.71, −0.32) and −0.45 (95%CI: −0.62, −0.29) at the lumbar spine and femoral neck, respectively, in adults with IBD [152]. This was coupled with an increased global risk of fracture in adults with IBD compared to controls (RR: 1.38 (95%CI: 1.11, 1.73)) [152]. In another population-based study, a higher likelihood of osteoporosis in IBD compared to healthy age and sex-matched controls was reported (OR: 1.47 (95%CI: 1.2, 1.78)) [146]. Even in studies with no overall deficits in areal bone density Z-scores in IBD, the prevalence of osteopenia remains high at 25−35% [148,149,150]. In adults with quiescent CD and established osteopenia (n = 23), bone biopsy histomorphometry highlighted an overall reduction in bone formation activity and associated trabecular thinning to be the primary pathogenic cause of skeletal deficits [153]. Similar findings of reduced bone formation compared to healthy controls were reported in another historic bone histology study of adults with IBD and established osteoporosis (n = 19) [154].

Imaging assessments of skeletal macro and microstructure is less common in adults compared to paediatric IBD. Two HR-pQCT studies reported deficits in trabecular microarchitecture, volumetric bone density, and cortical geometry, in young [144] and middle-aged [145] adults with IBD. Conversely, we recently reported no deficits in trabecular microarchitecture or cortical geometry in a cohort of young adults with mild or inactive childhood-onset CD compared to healthy age and sex-matched controls, using high-resolution MRI [5]. Our data suggest that children with CD may be able to recover any skeletal deficits incurred earlier in the disease course with good disease management using contemporary management strategies [155]. Such improvements have been previously reported in adults with strictly treated CD who had increased bone density at long-term follow-up [87]. Conversely, DXA [59,156] and HR-pQCT [144] studies reported low area bone density and microarchitectural parameters in cohorts including all or some young adults with childhood-onset IBD. In contrast to our recent published study, most published studies represent patients of a previous era in terms of disease management often with a long duration of exposure to high-dose glucocorticoids. Further prospective, high-resolution imaging studies are required to understand the progression of micro and macrostructural characteristics of bone in contemporary IBD cohorts especially in relation to nutritional and newer therapeutic strategies.

## 4. Future Directions

The persistence of musculoskeletal deficits in children and adults with IBD, particularly CD, despite advances in disease management and pharmacological therapies, highlights the need for research into effective therapeutic interventions which may include nutritional, lifestyle and pharmacological approaches. Research into understanding the patients who are at greatest risk of poor musculoskeletal outcomes using a genetic and epigenetic approach may allow the introduction of personalised therapies in the future.

In adults with IBD treated with glucocorticoid, the use of anti-resorptive (i.e., osteoclast targeting) bisphosphonate therapy is associated with attenuated bone loss and reduced fracture risk compared to non-treated controls [157]. In the clinical setting, the use of intravenous bisphosphonates is recommended following identification of fragility fracture (i.e., low trauma long bone fracture or vertebral fracture) [158]. Intravenous bisphosphonates are, however, associated with first dose acute reaction side effects which may be commoner and more severe in those with chronic disease and those treated with glucocorticoid. Newer anti-resorptive therapy using monoclonal antibodies targeting RANKL (Denosumab), has been shown to improve bone outcomes better than classic bisphosphonate therapy [159], is approved for use in glucocorticoid-induced osteoporosis, and is not associated with first dose reactions. Inhibition of RANKL may have an additional impact on skeletal muscle with early clinical data showing a reduction in the risk of falls and improved markers of sarcopenia in elderly patients [160]. Additionally, Denosumab was recently found to reduce colonic expression of pro-inflammatory cytokines and modulate disruption of the microbiome in a murine model of colitis mimicking CD [161], suggesting a potential role for RANKL in disease pathogenesis. Despite this, Denosumab remains relatively unexplored in IBD and further investigation of its impact on bone, muscle and disease outcomes in pre-clinical and human IBD studies is required. In addition, the safety of the use of this strategy especially upon discontinuation of therapy should also be considered in research studies.

Particularly in younger IBD patients at risk of musculoskeletal morbidity, the role of non-pharmacological approaches like nutritional support and exercise therapy as an adjunct to current IBD treatment strategies should be explored in high-quality clinical trials. Future research on how these interventions can be incorporated into the routine clinical care of IBD patients is also important to facilitate maximum impact on health and disease outcomes.

## 5. Conclusions

Multiple factors which include the underlying disease activity, nutritional issues, and use of glucocorticoids can impact musculoskeletal outcomes via diverse mechanisms in children and adults with IBD. Many patients despite good IBD symptom control have persistent deficits in muscle and bone health in published cohorts. Research into strategies to improve future musculoskeletal outcomes in IBD focusing on optimising disease targets beyond symptoms, plus improving nutritional status and beneficial lifestyle measures should now be prioritised.

## Figures and Tables

**Table 1:** Summary of major clinical bone imaging studies in paediatric IBD.

Author (year) Country	Design	Participants (N) Age (years)	Methods	Current or Previous Glucocorticoids (%)	Bone/Muscle Findings	Comments
Altowati et al. (2018) [123] Scotland	Prospective cohort	19 CD 14.9 (11.2, 17.2)	pQCT non-dominant radius and tibia DXA TB and LS	Current: 47%.	↓ Trabecular vBMD ↔ Cortical vBMD ↓ Cortical thickness ↓ Muscle CSA (Radius only) ↓ TB and LS aBMD	No improvement in bone or muscle outcomes after 12 months anti-TNF-α. IGF-1 improved only in those with low levels at baseline.
Ward et al. (2017) [6] Canada	Cross-sectional cohort	73 CD 13.9 (7, 17.7)	pQCT left tibia DXA TB and LS VF radiograph	Current: 64%	↓ Trabecular vBMD ↑ Endosteal and ↓ Periosteal circ. ↓ Cortical thickness and Muscle CSA ↓ LS aBMD, BMAD and TB BMC 1 VF	Newly diagnosed <35 days of initial therapy. 90% moderate-to-severe disease.
Maratova et al. (2017) [86] Czech Republic	Cross-sectional cohort	70 IBD (53 CD) 14.2 (IQR 12.7, 16.1)	pQCT non-dominant tibia VF radiograph	Current: NR Ever: 23% (within previous 12 months)	↓ Trabecular vBMD ↓ Cortical Thickness ↑ Cortical vBMD and SSI 1 VF; ↓ vertebral height in 27%.	Height adjusted pQCT Z-scores. All remission or mild disease.
Griffin et al. (2015) [124] USA	Prospective cohort	74 CD 14 (5, 21)	pQCT left tibia	Current: 32%	↓ Trabecular vBMD ↔ Cortical vBMD ↓ Muscle CSA ↑ Endosteal and ↓ Periosteal circ.	Infliximab induction cohort. 50% severe disease. Trabecular vBMD improved but still low at 12 months.
Pichler et al. (2015) [141] Austria	Retrospective	18 CD 14.4 (5.3, 19.1)	DXA LS	Current: 61%	↓ LS aBMD and BMAD	No change in aBMD or BMAD after 12-months anti-TNF-α.
Laakso et al. (2012) [142] Finland	Cross-sectional cohort	80 IBD (28 CD) 14.9 (5.1, 20.1)	DXA TB, LS, TH and VF	Current: 30% Ever: 81%	↓ TB & LS aBMD ↔ TH aBMD 16 VF (across 9 participants)	No differences between CD and UC. DXA Z-scores adjusted for chronological age and bone age. Large within-group variation in disease duration and cumulative GC.
Werkstetter et al. (2013) [121] Germany	Prospective cohort	10 CD 13.7 (10.6, 17.7)	pQCT non-dominant radius	GC naïve	↔ Trabecular and cortical vBMD ↓ Muscle CSA After 12 weeks: Trabecular vBMD and Muscle CSA ↑, cortical vBMD ↓. No further changes at 52 weeks.	Newly diagnosed cohort. All subsequently treated with 8 weeks EEN induction therapy. Height adjusted pQCT Z-scores. Short-term improvements in bone turnover and IGF-1, no further change at 52 weeks.
Ward et al. (2010) [14] Canada	Cross-sectional cohort	20 IBD (17 CD) 14.7 (8.4, 17.7)	Iliac biopsy histomorphometry DXA TB and LS VF radiograph	GC naïve	↔ Trabecular bone volume ↑ Trabecular number ↓ Cortical thickness ↓ LS aBMD and BMAD ↓ TB lean mass No evidence of VF	Newly diagnosed cohort. DXA lean mass Z-scores adjusted for height.
Bechtold et al. (2010) [12] Germany	Cross-sectional cohort	143 IBD (98 CD) New IBD: 13 ± 3.3 Established IBD: 14.7 ± 3.4	pQCT non-dominant radius	New IBD: GC naïve Established IBD: NR	↓ Trabecular vBMD ↑ Cortical vBMD ↓ Bone and Muscle CSA	All IBD vs controls. Muscle CSA Z-score lower in newly diagnosed vs established disease. GC history did not influence results.
Dubner et al. (2009) [122] USA	Prospective cohort	78 CD 12.7 ± 2.8	pQCT left tibia	GC naïve	↓ Trabecular vBMD ↔ Cortical vBMD ↓ Muscle and Fat CSA ↑ Endosteal and ↓ Periosteal Circ. ↓ Section modulus (Zp)	Newly diagnosed cohort. pQCT geometry Z-scores adjusted for tibia length. 84% growth failure. Persistent deficits in trabecular vBMD and muscle CSA at 12-months follow-up.
Sylvester et al. (2007) [13] USA	Prospective cohort	58 CD 13 ± 3	DXA TB and LS	56% GC during follow-up.	↓ TB & LS aBMD. No change in Z-scores at 2-year follow-up.	Newly diagnosed cohort. Bone age delay ~1 year in CD. DXA adjusted for bone age. Elevated serum IL-6 associated with low aBMD. TB aBMD positively correlated with IGF-1.
Burnham et al. (2005) [58] USA	Cross-sectional cohort	104 CD 15.4 ± 4.3	DXA TB	Current: 11% Ever: 90%	↓ TB BMC in sex, height and puberty adjusted models. ↔ TB BMC when + adjustment for lean mass.	DXA adjusted for bone size. Low BMI Z-score associated with low bone mass. GC history is not correlated with growth or bone outcomes.
Gupta et al. (2004) [143] Canada	Prospective cohort	123 IBD (82 CD) 11.8 ± 2.6	DXA LS	NR	↓ LS aBMD Z-score in CD ↔ LS aBMD Z-score in UC	Incident cohort. Age-adjusted DXA Z-scores. Not adjusted for body size.

N, number; CD, Crohn’s disease; UC, ulcerative colitis; IBD, inflammatory bowel disease; GC, glucocorticoids; aBMD, areal bone mineral density; vBMD, volumetric bone mineral density; BMAD; bone mineral apparent density; BMC, bone mineral content; CSA, cross-sectional area; DXA, dual-energy x-ray absorptiometry; pQCT, peripheral quantitative computed tomography; TB, total body; TH, total hip; LS, lumbar spine; VF, vertebral fracture; Circ., circumference ↑, high; ↓, low; ↔, not different.

**Table 2:** Summary of major bone imaging studies in adults with IBD.

Author (year) Country	Design	Participants (N) Age (years)	Methods	Current or Previous Glucocorticoids (%)	Bone/Muscle Findings	Comments
Steell et al. (2020) [5] Scotland	Cross-sectional cohort	27 CD 23.2 (18, 36)	MRI distal femur	Current: 4% Ever: 78%	↔ Trabecular microarchitecture ↔ Cortical geometry ↓ Muscle CSA ↑ Muscle fat	Childhood-onset cohort. Comparison w/ age and sex-matched controls. GC exposure negatively associated w/ trabecular bone volume. Muscle CSA positively associated w/ trabecular bone volume.
Sigurdsson et al. (2020) [59] Sweden	Cross-sectional cohort	94 IBD (29 CD) 21.8 (18.3, 27.7)	DXA TB, LS, FN, SMI	Current: NR Ever: 93%	↓ TB, LS, FN aBMD Z-scores in male CD and UC. ↓ SMI Z-score in male CD, not UC. ↔ TB, LS, FN aBMD and SMI Z-scores in female CD and UC.	Childhood-onset cohort. DXA not adjusted for body size. Myopenia and myopenic-obesity associated with low aBMD at all sites.
Pepe et al. (2018) [144] Switzerland	Cross-sectional cohort	102 IBD (75 CD) 23.1 ± 5.8	HR-pQCT non-dominant distal radius and tibia DXA LS, FN, PF and Radius, VF	Current: 17%. Ever: NR	↓ aBMD at all sites. ↓ Total and trabecular vBMD ↓ Trabecular number and Cortical thickness at the tibia. ↑ Trabecular separation at radius and tibia 5 VF	Includes paediatric and adult IBD. Fractured IBD had lower total and trabecular vBMD and trabecular thickness vs non-fractured.
Haschka et al. (2016) [145] Germany	Cross-sectional cohort	98 IBD (59 CD) 42.8 (IQR 30, 54)	HR-pQCT dominant ultra-distal radius	Current: 14%	↓ Cortical vBMD in CD and UC. ↓ Cortical thickness in CD and UC. ↓ Cortical area in CD, not UC. ↓ Total and trabecular vBMD in CD, not UC. ↓ Trabecular bone volume and thickness in CD, not UC.	Poorer vBMD and structural outcomes in CD vs UC. 50% history of chronic high-dose GC. Low BMD, female sex, and lack of remission associated with a low cortical area.
Targownik et al. (2013) [146] Canada	Retrospective	1230 IBD (719 CD) 49 (IQR 38, 57)	DXA LS, FN, TH	Current: 23%	↓ aBMD T-scores at all sites in CD, not UC.	Increased risk of osteoporosis for CD vs controls in adjusted regression (OR: 1.47 (1.2, 1.78)). aBMD T-scores negatively associated with GC at all sites.
Leslie et al. (2008) [147] Canada	Prospective cohort	101 IBD (56 CD) 46.9 ± 15.5	DXA TB, LS and TH	Current: NR Ever: 60%	↓ LS and TH aBMD T-scores ↔ TB aBMD T-scores ↔ TB, LS and TH aBMD Z-scores	Mean LS aBMD T-score at the lower end of normal (−0.76 ± 1.2). Men lower Z-scores vs women at LS.
Bernstein et al. (2003) [148] Canada	Cross-sectional cohort	70 IBD (58 CD) 33 ± 7.4	DXA TB, LS, FN and TH	Current: NR Ever: 82%	↔ aBMD T-scores at all sites 25/70 (36%) osteopenic T-scores at ≥1 site.	‘Early onset’ IBD (diagnosed <20 years). CD & UC presented together. Manufacturer reference data. Low BMD associated with recent amenorrhea and low body weight.
Schoon et al. (2000) [149] Netherlands	Cross-sectional cohort	68 IBD (24 CD) 29.7 ± 10.4	DXA TB, LS and TH	Current: 33%	↔ aBMD Z-scores at all sites 18/68 (26%) osteopenic T-scores at ≥1 site.	Incident cohort <6 months from diagnosis. Mean T-scores not reported. 12.5% active disease at the time of DXA. Local controls for Z-scores (n = 68).
Robinson et al. (1998) [150] England	Cross-sectional cohort	117 CD 40.6 ± 13.3	DXA LS, PF	Current: 22% Ever: 86%	↔ aBMD Z-scores at all sites 34/117 (29%) osteopenic, 14/117 (12%) osteoporotic (Z-scores)	DXA age- and sex-adjusted Z-scores. aBMD negative association with GC history.
Ghosh et al. (1994) [151] Scotland	Prospective cohort	30 IBD (15 CD) 24 (14, 83)	DXA LS, right arm	Current: 47%	↓ LS & arm aBMD Z-scores in CD	Newly diagnosed cohort. No change in Z-scores after 12 months follow-up.

N, number; CD, Crohn’s disease; UC, ulcerative colitis; IBD, inflammatory bowel disease; GC, glucocorticoids; aBMD, areal bone mineral density; vBMD, volumetric bone mineral density; BMC, bone mineral content; SMI, skeletal muscle index; CSA, cross-sectional area; DXA, dual-energy x-ray absorptiometry; MRI, magnetic resonance imaging; pQCT, peripheral quantitative computed tomography; HR-pQCT, high resolution peripheral quantitative computed tomography TB, total body; TH, total hip; LS, lumbar spine; PF, proximal femur; FN, femoral neck; VF, vertebral fracture; Circ., circumference; IQR, inter-quartile range; OR, odds ratio; ↑, high; ↓, low; ↔, not different
